# User-friendly bioorthogonal reactions click to explore glycan functions in complex biological systems

**DOI:** 10.1172/JCI169408

**Published:** 2023-03-15

**Authors:** Xi Chen, Ajit Varki

**Affiliations:** 1 Department of Chemistry, UCD, Davis, California, USA.; 2 Departments of Medicine and Cellular & Molecular Medicine, Glycobiology Research and Training Center, UCSD, San Diego, California, USA.

## Chemical biology, an integration of multiple fields

When the Nobel Prizes were first created in 1901 to honor major progress in various domains of human knowledge, the awards for scientific progress could be roughly classified by levels of certainty, with physics and chemistry having apparently immutable laws in the known universe, and physiology and medicine having less precision. But bridging disciplines, such as organic chemistry, biochemistry, molecular biology, and cell biology, were still in their infancy, and the overarching principles of evolutionary biology were not yet established. We are now in an era wherein these previously distinct disciplines are overlapping and merging, and chemical biology (application of chemical techniques, analysis, and compounds to study and manipulate biological systems) is receiving much attention. Remarkable and practically relevant examples were recognized with the 2022 Nobel Prize in Chemistry, which was awarded to Morten Meldal and K. Barry Sharpless for the development of “click chemistry” and to Carolyn R. Bertozzi for the concept of “bioorthogonal chemistry.” Meldal and Sharpless developed fast-rate copper(I)-catalyzed azide-alkyne cycloaddition (CuAAC, referred to herein as click) reactions, which have found broad applications in various fields for different purposes (1). This brief Viewpoint focuses on Bertozzi’s groundbreaking bioorthogonal chemistry concept. Berozzi coined the term to refer to any chemical reaction that can occur inside of living systems without interfering with native biochemical processes (2) and led the advances in developing such reactions and applying them in vivo. These user-friendly chemical reactions, reagents, and tools are critical for exploring the functions and applications of target molecules, especially those in living systems, and they expand the applications of chemical biology to better understand and treat human disease.

## Glycans: essential but understudied biomolecules

Besides DNA, RNA, proteins, and lipids, the other major macromolecular components of living systems are carbohydrates (hereafter generically called glycans). All mammalian cells and extracellular matrices contain a high density of glycan biomolecules, including glycoproteins, glycolipids, proteoglycans, and polysaccharides (3). Such glycans are among the most abundant organic molecules in nature, are essential for all living organisms, and participate in most biological processes (4), but they are also the most challenging to study. Unlike DNA and RNA or proteins, which are genetically coded and produced by template-drive processes, there is no uniform “glycocode,” and glycan biosynthesis relies on coordinated efforts of different enzymes and subcellular compartments in a cell-, tissue-, and species-specific manner. Furthermore, glycans are the most diverse biomolecules, constructed with building blocks selected from a large collection of structurally different monosaccharides, connected with a variety of glycosyl linkages, with the possibility of multiple branches and disparity of molecular weights. Lack of proper tools and user-friendly synthetic strategies, as well as their dynamic structural changes in response to environmental variations, add to the difficulties in analyzing, studying, and elucidating the structures and functions of complex glycans, especially those in living organisms. Therefore, glycans are ideal targets for applying the user-friendly click chemistry and bioorthogonal reactions.

## Sialic acids as initial targets for the development of bioorthogonal reactions

Among monosaccharide building blocks of human glycans, sialic acids are common terminal units on the glycan portion of cell surface glycoproteins and glycolipids (5) and can be further decorated with a diverse array of additional modifications, including some added after the formation of glycosyl linkages. In the early 1990s, Werner Reutter and coworkers made critical advances in using modified sugar units as metabolic precursors to redesign cell surface glycan structures and influence their functions (6). Inspired by these pioneering studies (7), Bertozzi innovatively combined a living cell’s sophisticated synthetic capabilities and tolerance toward structural modification of metabolic molecules with a chemist’s ingenuity to deliver chemical handles, which are bioorthogonal to the cell environment, to the desired molecular targets. The resulting chemical handles can be selectively visualized or tagged in the cell environment rich with chemical functional groups. Initial proof-of-concept demonstration was showcased in feeding cultured mammalian cells with a ketone-containing *N*-acyl derivative of mannosamine, which was metabolically converted to ketone-containing sialic acids on glycoconjugates presented on cell surface. Conjugation of the ketone with biotin hydrazide led to selective tagging of cell surface sialic acids for fluorescent labeling or toxin delivery (8).

## Delivering hydrophilic glycans into cells

The trailblazing path to performing bioorthogonal reactions in living systems overcame several obstacles, resulting in improved metabolic precursor delivery, enhanced bioorthogonality of the reaction, and faster reaction rate. Monosaccharides are hydrophilic molecules, and some can be delivered from the extracellular environment into the cytosol by specific plasma membrane transporters in a controlled manner (concentration, equilibrium) or delivered via fluid-phase macropinocytosis to lysosomes (9). For some other monosaccharides and their modified derivatives or glycosides (10), none of these approaches work, but an efficient way to significantly enhance cellular uptake uses per-*O*-acetylated analogs. Upon cellular uptake via hydrophobic interactions with the plasma membrane lipid bilayer, the *O*-acetyl groups are readily removed from monosaccharides by nonspecific esterases in cytoplasm, providing unprotected monosaccharide derivatives that can be metabolically processed and incorporated into endogenous macromolecules.

## The Bertozzi-Staudinger ligation

**Figure 1:**
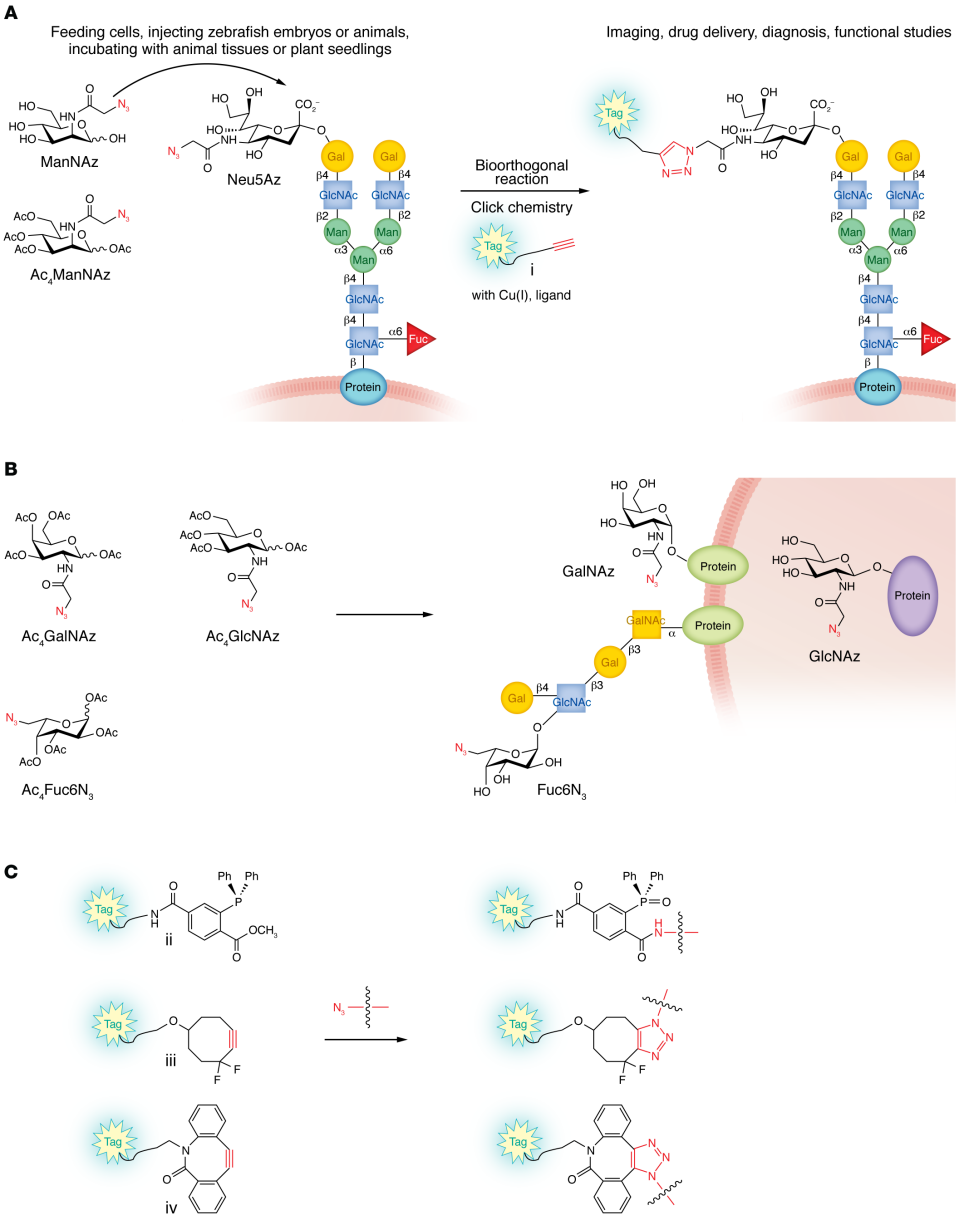
Metabolic glycoengineering and bioorthogonal reactions leading to tagging targets selectively for imaging, drug delivery, diagnosis, and functional studies. (**A**) Examples of using *N*-azidoacetylmannosamine (ManNAz) or per-*O*-acetylated ManNAz (Ac_4_ManNAz) as metabolic precursors of *N*-azidoacetylneuraminic acid (Neu5Az) are shown. The reaction of a metabolically engineered azido-containing sialic acid with a terminal alkyne-containing compound (i) by copper(I)-catalyzed azide-alkyne cycloaddition (CuAAC) in the presence of a tris(triazolylmethyl)amine-based ligand forms a triazole-linked product. ManNAz is taken up by cells, converted to activated Neu5Az, which is transferred to cell surface glycoproteins as an azido-containing derivative of the most common sialic acid, Neu5Ac. Per-*O*-acetylation of monosaccharides enhances their uptake by cells. *O*-Acetyl groups are removed by cytoplasmic esterases, and the azido-containing monosaccharides go through metabolic processes to present on the glycan components of glycoconjugates. CuAAC leads to visualizing glycan changes in live cells, tissues, or organisms and can be applied for drug delivery, diagnosis, and functional studies of the important roles of glycans. (**B**) Additional per-*O*-acetylated monosaccharide precursors that have been explored, such as per-*O*-acetylated *N*-azidoacetylgalactosamine (Ac_4_GalNAz), *N*-azidoacetylglucosamine (Ac_4_GlcNAz), and 6-azido-L-fucose (Ac_4_Fuc6N_3_). (**C**) Additional representative bioorthogonal reaction counterparts of azides, including novel triarylphosphine (ii), difluorinated cyclooctyne (iii), and biarylazacyclooctynone (BARAC) (iv) probes. Colors and shapes of monosaccharides follow the Symbol Nomenclature for Glycans (31).

The bioorthogonality of the reactions has been improved by replacing the ketone functionality on the sugar precursor with an azido group as the chemical handle. The latter can be installed easily by chemical derivatization of the sugar precursor, is a functional group absent from biosystems, and is small enough to be accommodated by metabolic processes. A novel triarylphosphine reagent (Figure 1C, ii) was designed to allow it to react with the azide (11) to form a stable covalent-linked adduct under physiological conditions. This “Bertozzi-Staudinger ligation” was applied to sialic acid using its per-*O*-acetylated *N*-azidoacetylmannosammine (Ac_4_ManNAz) precursor (11) (Figure 1A) and extended to per-*O*-acetylated *N*-azidoacetylgalactosammine (Ac_4_GalNAz) (12) and per-*O*-acetylated *N*-azidoacetylglucosammine (Ac_4_GlcNAz) (13) (Figure 1B). In addition to demonstrations in cell culture systems, such reactions have been successfully applied to live mice (2) and other model organisms. This work set the stage for more precise labeling of specific structures via designer azido sugars and engineered pathways (14).

## Overcoming other technical limitations

To overcome the limitations of using phosphine reagents (vulnerable to oxidation by air, low water solubility, slow reaction rate) as bioorthogonal detection reagents for azides and avoid the need of using toxic copper(I) as a catalyst for the click reactions initially developed by the Sharpless (15) and the Meldal (16) groups, the Bertozzi group pioneered the effort of developing strain-promoted alkyne probes, such as cyclooctyne (17), and its difluorinated analogs (DIFO; Figure 1C, iii). DIFO was used successfully with Ac_4_GalNAz precursor for imaging glycans in developing zebrafish (18). The reaction rate was further improved by developing a biarylazacyclooctynone reagent (BARAC; Figure 1C, iv) (19). These bioorthogonal reagents react with the azido groups introduced to biomolecules, including complex glycoconjugates, via strain-promoted azide-alkyne cycloaddition (SPAAC) reactions, which are also called copper-free click reactions.

With the advantage of their easy utilization, commercial availability, and the development of tris(triazolylmethyl)amine-based ligands, such as BTTAA (20), as copper(I)-stabilizing ligands that can enhance the reaction efficiency of CuAAC and decrease the amount of toxic copper(I) needed for the reaction, CuAAC reactions (Figure 1A, i) have now been broadly adopted for metabolic glycoengineering and bioorthogonal reactions for cell-based studies. BTTAA has been found to be the most efficient that allowed the use of copper(I) catalyst at a concentration that does not cause significant toxicity to cells (20). Tris(triazolylmethyl)amine-based ligand reagents are also now commercially available, making the bioorthogonal strategy readily accessible to the broad community. With CuAAC, per-*O*-acetylated 6-azido-L-fucose (Ac_4_Fuc6N_3_; Figure 1B) has been used as the metabolic precursor for probing cell surface fucosylation with a terminal alkyne-containing CuAAC reaction counterpart (21).

## More bioorthogonal pairs

Adding to the efficient bioorthogonal tool set that has been applied with metabolic glycoengineering is another type of bioorthogonal pair of terminal alkenes and 1,2,4,5-tetrazines, which are ligated together by inverse-electron-demand Diels-Alder reaction (22). Strain-promoted inverse-electron-demand Diels-Alder reactions between 1,2,4,5-tetrazines and *trans*-cyclooctenes (23) have also been designed for local drug activation to selectively release chemotherapeutic drugs, such as doxorubicin, at the tumor site to generate enhanced antitumor responses (24).

## The revolution continues, with wide-ranging implications

The Nobel Prize announcement for click chemistry touched on its wide-ranging implications (25), stating that “The two concepts of click chemistry and bioorthogonal chemistry have had a tremendous impact on Chemistry and its neighboring sciences. The discoveries of CuAAC, SPAAC, and other related reactions addressed a significant unmet need, and spurred intense activity across many different areas” and “click chemistry and bioorthogonal chemistry [have] been used in the development of enzyme inhibitors and receptor ligands, pharmaceuticals (anticancer agents, antimicrobials, etc.), herbicides, photostabilizers, diagnostics and sensing elements, corrosion retardants, brightening agents, biomacromolecule conjugates, tissue regeneration matrices, and various macromolecular materials (gels, polymers, etc.), as well as in the mapping of complex biological processes.”

## Further improvements in metabolic glycoengineering

As with many major advances, improvements are still needed at several fronts. Investigations using metabolic glycoengineering strategies have been so far mainly targeting glycoproteins. Nevertheless, efficient methods for identifying the underlying structures that are metabolically tagged are still lacking. Glycolipids (26), another important class of glycoconjugates, are additional attractive targets. In general, competition of the endogenous native unmodified monosaccharides can lower the efficiency for incorporating the modified monosaccharides. For functional research studies, the efficiency of metabolic glycoengineering can be improved by the use of genetically engineered cells (27) in which one or more key enzymes that are involved in divergent pathways are removed. Although it increases the efficiency of cellular uptake, per-*O*-acetylation of unnatural sugars has been shown to nonspecifically react with protein cysteines via nonenzymatic *S*-glyco-modifications, which may lead to false-positive results (28). In addition, caution needs to be taken when interpreting the results of feeding cells or animals with modified biomolecules, because structural modifications, although small and tolerable by biosystems, will undoubtedly alter molecular interactions and change the presentation of native molecules.

## Potential immunological issues and advantages

For consideration of potential clinical applications of bioorthogonal chemistry, immune reactions against products of in vivo ligation could potentially occur when repeatedly used in the same individual. Such events can also be used as an advantage, to enhance the immunogenicity of weak immunogens (29), such as glycans. While further adaptations of biorthogonal chemistry are needed, the fundamental concept and the remarkable toolbox developed allow us to bring glycans out of the shadows and into the mainstream of biology. We look forward to seeing continued advances in the years to come.

## Renewed Nobel Prize recognition for studies of glycans

In closing, it is notable that, consistent with the prominence of glycans in nature, the first seven decades of the 20th century saw many Nobel Prizes awarded for chemical, biological, and biochemical studies related to glycans; these include Fischer (Chemistry, 1902, monosaccharide synthesis and structures); Buchner (Chemistry, 1907, cell-free fermentation of carbohydrates); Meyerhof (Physiology or Medicine, 1922, lactic acid formation from carbohydrates; Landsteiner (Physiology or Medicine, 1930, human blood groups); Haworth (Chemistry, 1937, composition and structure of various forms of sugar, starch, and cellulose); Carl and Gerty Cori (Physiology or Medicine, 1947, glycogen and glucose metabolism); and Leloir (Chemistry, 1970, sugar nucleotides). After a 50-year gap, during which the molecular biology of DNA, RNA, and proteins dominated and glycans languished in obscurity, the Nobel committee has once again given attention to this “dark matter of the biological universe” (30).

## Version 1 03/15/2023

Electronic publication
